# Antigenic changes in influenza A(H3N2) driven by genetic evolution: Insights from virological surveillance, EU/EEA, week 40/2023 to week 9/2024

**DOI:** 10.2807/1560-7917.ES.2024.29.50.2400395

**Published:** 2024-12-12

**Authors:** Eeva K Broberg, Maja Vukovikj, Olov Svartström, Iris Hasibra, Maximilian Riess, Angeliki Melidou, Members of the ERLI-Net network, Members of the ERLI-Net network, Sarah Denayer, François E Dufrasne, Neli Korsun, Ivelina Trifonova, Goranka Petrović, Irena Tabain, Helena Jiřincová, Timotej Šúri, Amanda Bolt Botnen, Ramona Trebbien, Johanna Kristina Tamm, Regina Russanova, Niina Ikonen, Erika Lindh, Vincent Enouf, Laurence Josset, Marianne Wedde, Ralf Dürrwald, Georgia Gioula, Mary Emmanouil, Brynja Ármannsdóttir, Guðrún Erna Baldvinsdóttir, Simona Puzelli, Marzia Facchini, Svajune Muralyte, Monika Maconkaite-Tekoriene, Anke Wienecke-Baldacchino, Trung Nguyen, Graziella Zahra, Jackie Melillo, Adam Meijer, Ron Fouchier, Andreas Rohringer, Karoline Bragstad, Lidia B. Brydak, Ewelina Hallmann, Raquel Guiomar, Ana Paula Rodrigues, Mihaela Lazar, Rodica Popescu, Edita Staroňová, Elena Tichá, Vesna Šubelj, Katarina Prosenc, Francisco Pozo, Inmaculada Casas, Tove Samuelsson Hagey, Neus Latorre-Margalef

**Affiliations:** 1 European Centre for Disease Prevention and Control (ECDC), Stockholm, Sweden; 2 World Health Organization (WHO) Regional Office for Europe, Copenhagen, Denmark; 3 Members of the ERLI-Net who contributed virus detection and/or characterisation data or were involved in weekly surveillance activities are listed under Collaborators (affiliations at the end of the article)

**Keywords:** influenza, surveillance, Europe, genetic

## Abstract

**Background:**

During the 2023/24 influenza season in the European Union/European Economic Area (EU/EEA), influenza viruses A(H1N1)pdm09, A(H3N2) and B/Victoria viruses were co-circulating.

**Aim:**

We aimed to describe the circulating influenza viruses by (sub)type, genetic clade, antigenic group and antiviral susceptibility in that season in the EU/EEA.

**Methods:**

We collected surveillance data from EU/EEA countries through weekly submissions to The European Surveillance System (TESSy). Data were submitted in strain-based format for weeks 40/2023 to 9/2024.

**Results:**

Twenty-nine EU/EEA countries reported 154,718 influenza virus detections (primary care sentinel and non-sentinel combined), of which 97% (150,692) were type A and 3% (4,026) were type B. Of the subtyped influenza A viruses, 30,463 (75%) were influenza A(H1)pdm09 and 10,174 (25%) were influenza A(H3). For 809 (20%) of the type B viruses, the lineage was determined; all were B/Victoria/2/87 lineage, and none were B/Yamagata/16/88 lineage. Genetic diversification of seasonal influenza viruses continued, and clade 5a.2a of A(H1N1)pdm09, 2a.3a.1 of A(H3N2) and V1A.3a.2 of B/Victoria-lineage viruses dominated. Of the A(H3N2) 2a.3a.1 viruses, 23% were antigenically distinct from the 2023/24 vaccine virus.

**Conclusion:**

The 2023/24 influenza season was characterised by co-circulation of different influenza (sub)types, antigenically similar to the components recommended for the 2023/24 northern hemisphere vaccine, A/Victoria/4897/2022 (egg-based) and A/Wisconsin/67/2022 (cell culture- or recombinant-based). However, genetic diversification of the viruses continued. The World Health Organization’s vaccine recommendations for the northern hemisphere 2024/25 season were updated to include a new A(H3N2) component, while maintaining the current A(H1N1)pdm09 and B/Victoria components.

**Key public health message**

**What did you want to address in this study and why?**

Influenza virus vaccine components need to be updated each year to match the circulating influenza viruses. We wanted to describe influenza viruses circulating in the EU/EEA countries during 2023/24 influenza season to understand how the circulating viruses compare to the vaccine components.

**What have we learnt from this study?**

The genetic diversification of all seasonal influenza viruses continued. Despite this, influenza A(H1N1)pdm09- and influenza B/Victoria-lineage viruses were well covered by the vaccine strains. However, influenza A(H3N2) viruses did not fully match with the vaccine strain. B/Yamagata-lineage viruses were not detected at all through current surveillance systems.

**What are the implications of your findings for public health?**

Our study showed that in Europe, as elsewhere in the world, it was important to have an update to the vaccine component of the influenza A(H3N2) viruses as the genetic diversification of those viruses has made these viruses different from the previous vaccine component.

## Background

Continuous virological surveillance of influenza virus strains is of great importance as seasonal influenza viruses evolve constantly both genetically and antigenically and thus vaccine components need regular evaluations. The influenza virus characterisation data from countries in the European Union and European Economic Area (EU/EEA) provide a key component of the World Health Organization (WHO) European Region data submitted to the WHO vaccine composition consultation on a semiannual basis.

This article discusses the results of 2023/24 influenza season in the context of the WHO vaccine composition recommendations for the northern hemisphere (NH) 2024/25 influenza season [1].

## Methods

We summarise influenza virological surveillance data in the EU/EEA, for the reporting period week 40/2023 through 9/2024, as notified by national influenza centres (NICs) or national reference laboratories (NRLs) by 7 March 2024 to The European Surveillance System (TESSy) hosted at the European Centre for Disease Prevention and Control (ECDC). The data period embraced 22 weeks. The data sources and methods have been described earlier [2] and were here supplemented with additional influenza detection reporting through a new integrated respiratory virus surveillance aggregate reporting protocol [3] and implementation of Nextstrain subclade naming [4]. In short, the detection of influenza A and B viruses, subtyping of influenza A(H1N1)pdm09 and A(H3N2) viruses and, in some instances, type B lineage determination was performed with real-time RT-PCR techniques. Weekly detection and testing data by country were reported to TESSy in aggregate format by week of sampling. The NICs and NRLs conducted antigenic characterisation of viruses through haemagglutination (HA) inhibition (HI) assay, using strain-specific post-infection ferret antisera raised against vaccine viruses and reference viruses raised by the laboratories on their own, or provided by the WHO Collaborating Centres (CC) in Atlanta, United States or London, United Kingdom. A virus isolate was considered antigenically similar to a reference virus if the HI titre with the respective post-infection ferret antiserum differed by no more than fourfold (usually a decrease), in a twofold dilution series, from the HI titre of the antiserum with the reference virus itself. To consider an isolate antigenically different from a reference virus, the HI titre had to show a decrease of eightfold or more. For antigenic characterisation of influenza A(H3N2) viruses, some NICs conducted HI assays in the presence of oseltamivir, to prevent haemagglutination by the N2 neuraminidase, and/or performed virus neutralisation assays. Antigenic characterisations are reported to TESSy under the different representative influenza virus categories in strain-based format. In addition, ‘not attributed to category’ was available for each subtype and lineage to accommodate viruses that either did not match one of the preset major antigenic groups or did not yield a conclusive HI assay result.

The NICs and NRLs also conducted genetic characterisation of viruses through sequencing, often directly on clinical specimens. We downloaded all seasonal influenza HA sequences for influenza A(H1N1)pdm09, A(H3N2) and B/Victoria in the 2023/24 season from the EpiFlu database of GISAID [5]. We used an ECDC in-house programme to process the sequence data for each subtype separately as follows: all entries in TESSy, reported with an HA sequence and available on GISAID, were matched with the downloaded GISAID data, keeping entries in TESSy with a matching GISAID isolate identification number or influenza gene segment sequence accession number (complemented with a few cases of isolate name matches) number and extracting the sequences of those matches into a separate file. We excluded HA sequences in cases of unreleased sequences, errors in the accession number or a mismatch between the name of the virus in the TESSy report and GISAID. An HA sequence length limit of at least 900 bp was also required. Alignment was performed using mafft v7, first aligning the reference sequences and then adding the available test sequences, and the alignment was trimmed to include only the HA1 coding region. We used RAxML v8.2.7 to construct a phylogenetic tree using 10 bootstraps and a maximum likelihood search. The tree was rooted on the oldest reference sequence using treesub, and PAML baseml v4.9f was used to perform ancestral reconstruction of the HA1 sequences for all internal nodes of the tree. We used Treesub to annotate the tree branches with amino acid substitutions, based on the root sequence. The nodes were coloured according to month and the tree was exported in nexus format. We retrieved clades for the references by querying their HA sequences on Nextclade. The clades of the sequences were determined by comparison with the references in the phylogenetic tree. The trees were edited and annotated using FigTree and Inkscape. We used HA amino acid sequence alignments to inspect amino acid substitutions in Bioedit, Flusurver and Nextclade. We compared the circulation patterns of influenza viruses in Europe to the ones circulating globally through Nextstrain real-time tracking of influenza virus evolution for the same reporting period as our analysis [6].

Data on susceptibility to neuraminidase inhibitor (NAI) antiviral agents were produced by the NICs using genotypic (limited SNP detection by RT-PCR or pyrosequencing, or partial or full NA gene sequence analysis) and/or phenotypic analysis (drug-specific IC50 determination), and the results were reported to TESSy. For genotypic analysis, susceptibility was determined by the reported amino acid substitutions associated with reduced/highly reduced inhibition (RI/HRI) by the NAIs oseltamivir or zanamivir [7]. Phenotypic susceptibility was assessed by determining half-maximal inhibitory concentration (IC50) values, representing the concentration of oseltamivir or zanamivir needed to inhibit viral neuraminidase activity by 50%. For influenza A viruses, inhibition was classified as normal inhibition (NI) if a reported value was a < 10-fold increase above the median IC50 value after removal of obvious outliers. Reduced inhibition required a 10 to 100-fold increase above the median IC50 and HRI required > 100-fold above the median IC50. For influenza B viruses, the corresponding values were: < 5-fold increase above median (NI); 5–50-fold increase above median (RI) and > 50-fold increase above median (HRI) [8]. The NICs calculated median values and fold-changes by virus (sub)type, antiviral drug and IC50 assay method. The submitting laboratories reported their own interpretation of the genotypic and phenotypic assessments as NI, RI or HRI to TESSy, and the same with the prefix ‘AA’ for genotypic assessments. If no assessment was done, ‘not applicable’ (NA) was reported and if genotypic interpretation was not possible, that was reported separately as ‘amino acid interpretation not possible’ (AAINP). We used these assessments of the submitting laboratories for the calculations in this analysis. If both phenotypic and genotypic results were reported, phenotypic results took precedence. If the analysis team disagreed with the interpretation of the country, we contacted the country and received their agreement to update the interpretation.

Polymerase acidic protein (PA) amino acid substitutions associated with reduced baloxavir marboxil susceptibility were defined using WHO reference guidance [9]. The IC50 fold-change threshold for identifying a reduced susceptible virus was set at 3.

## Results

### Detections

**Figure 1:**
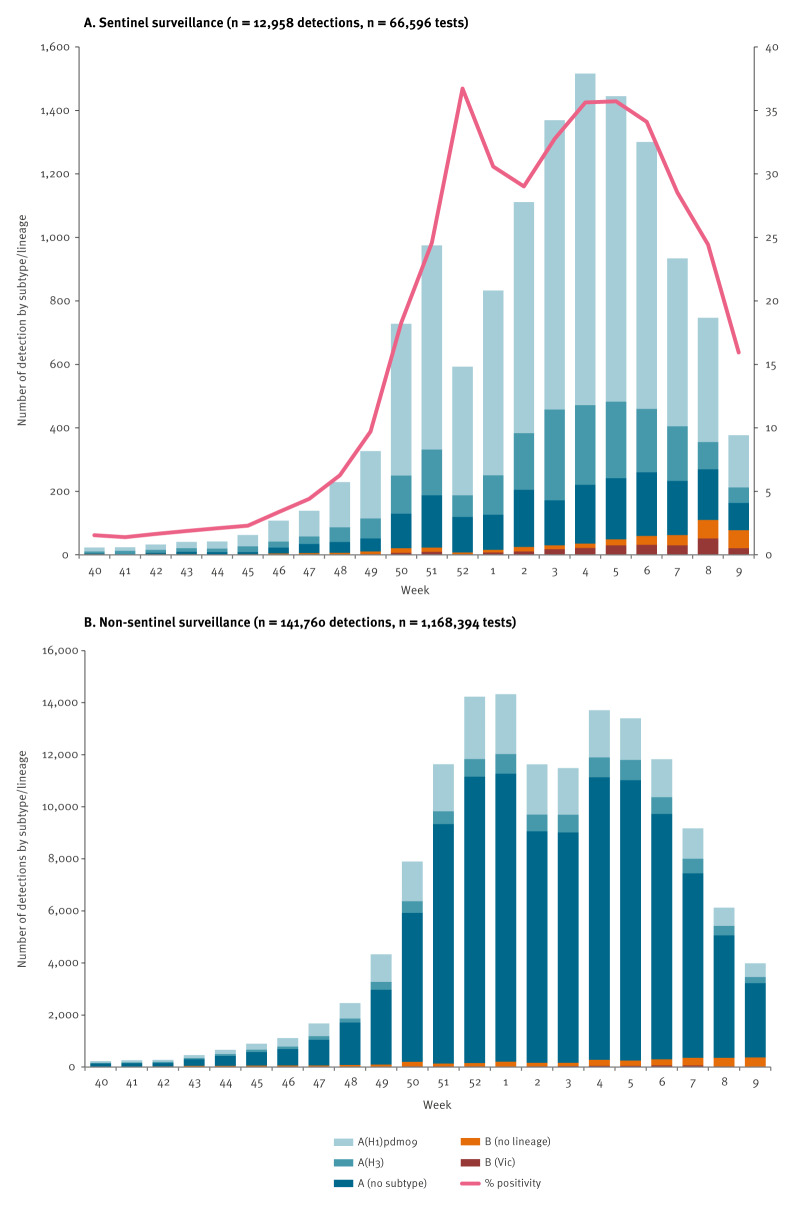
Number of detections by subtype and percentage positive of all tested, by week, EU/EEA, weeks 40/2023–9/2024

EEA: European Economic Area; EU: European Union.

In the reporting period, 29 EU/EEA countries reported 154,718 influenza virus detections (primary care sentinel and non-sentinel combined), of which 150,692 (97%) were type A and 4,026 (3%) were type B virus (Table 1, Figure 1). In total, 1,234,990 tests were performed (Table 1, Figure 1). 

**Table 1:** Influenza virus detections in sentinel and non-sentinel source specimens by type and subtype cumulatively, EU/EEA, weeks 40/2023–9/2024 (n = 154,718)

	Sentinel		Non-sentinel	
Detections by virus (sub)type	n	%	n	%
**Influenza A**	**12,397**	**95.7^a^**	**138,295**	**97.6^a^**
A(H1)pdm09	8,296	79.5**^b^**	22,167	73.4**^b^**
A(H3)	2,136	20.5**^b^**	8,038	26.6**^b^**
A not subtyped	1,965	NA	108, 090	NA
**Influenza B**	**561**	**4.3^a^**	**3,465**	**2.4^a^**
B/Victoria lineage	256	100.0**^c^**	553	100.0**^a^**
B/Yamagata lineage	0	0**^c^**	0	0**^c^**
B Unknown lineage	305	NA	2,912	NA
**Total detections** **(total tested)**	**12,958** **(66,596)**	**19.5**	**141,760** **(1,168,394)**	**12.1**
Antigenic characterisations	n		%	
**Influenza A**				
**A(H1)pdm09**	**512**		**100.0**	
5a.2a A/Sydney/5/2021-like^d^	262		51.2	
5a.2a.1 A/Victoria/4897/2022-like^e,f^	248		48.4	
5a.2a.1 A/Wisconsin/67/2022-like^e,f^	2		0.4	
**A(H3)**	**103**		**100.0**	
2a A/Darwin/9/2021-like^d,e,g,h^	76		73.8	
2a.3a.1 A/Thailand/8/2022-like^f^	24		23.3	
Not categorised	3		2.9	
**Influenza B**				
**B/Victoria lineage**	**60**		**100.0**	
V1A.3a.2 B/Austria/1359417/2021-like^d,e,f,g,h^	58		96.7	
Not categorised	2		3.3	
Phylogenetic analysis^i^	n		%	
**Influenza A**				
**A(H1)pdm09**	**1,815**		**100.0**	
5a.2a (C.1)	1,029		56.7	
5a.2a + T216A (C.1.7)	54		3.0	
5a.2a.1 (C.1.1)	22		1.2	
5a.2a.1 + T216A (C.1.1.1)	710		39.1	
**A(H3)**	**639**		**100.0**	
2a.3a (G.1.3.1)	10		1.6	
2a.3a.1 (J)	50		7.8	
2a.3a.1 + I25V (J.1)	212		33.2	
2a.3a.1 + N122D, K276E (J.2)	346		54.1	
2a.3a.1 Q173R, K276E (J.4)	20		3.1	
2a.3b (G.1.3.2)	1		0.2	
**Influenza B**				
**B/Victoria lineage**	**90**		**100.0**	
V1A.3a.2 (C.2)	1		1.1	
V1A.3a.2 (C.3)	2		2.2	
V1A.3a.2 (C.5)	9		10.0	
V1A.3a.2 (C.5.1)	35		38.9	
V1A.3a.2 (C.5.6)	14		15.6	
V1A.3a.2 + E128G (C.5.7)	29		32.2	

EEA: European Economic Area; EU: European Union; NA: not applicable; WHO: World Health Organization.

^a^ Proportion of influenza A or B type.

^b^ Proportion of influenza A subtypes.

^c^ Proportion of B virus lineages.

^d^ WHO-recommended vaccine virus for the 2023 southern hemisphere influenza season.

^e^ WHO-recommended vaccine virus for the 2023/24 northern hemisphere influenza season.

^f^ WHO-recommended vaccine virus for the 2024 southern hemisphere influenza season.

^g^ WHO-recommended vaccine virus for the 2022 southern hemisphere influenza season.

^h^ WHO-recommended vaccine virus for the 2022/23 northern hemisphere influenza season.

^i^ For the phylogenetic analysis section, subclades in brackets are defined through Nextstrain [4].

In panel A, the red line shows the percentage positivity for influenza detections per week.

Of the subtyped influenza A viruses, 30,463 (75%) were influenza A(H1)pdm09 and 10,174 (25%) were influenza A(H3). Of the 4,026 reported influenza type B viruses, the lineage for 809 (20%) was determined, with all viruses belonging to the B/Victoria/2/87 lineage. No B/Yamagata/16/88 lineage virus was reported (Table 1, Figure 1). All EU/EEA countries experienced an influenza A(H1)pdm09 virus-dominated season with similar temporal patterns of (sub)type distribution (data not shown).

### Genetic characterisation

Within the reporting period, 2,567 viruses (2% of all 154,718 surveillance source detections; 6% (766 sequences) of 12,598 sentinel source detections) from 15 countries were reported with sequence identifiers, of which 2,544 sequences could be retrieved and included in the phylogenetic analysis. The main contributors of sequences were: Spain (n = 495; 20%), the Netherlands (n = 393; 15%), Germany (n = 389; 15%) and Norway (n = 341; 13%), with other countries contributing between <1% and 7% (range: 5–179).

Of the 1,815 influenza A(H1N1)pdm09 viruses with available sequences, 1,083 (60%) belonged to clade 5a.2a, while 732 (40%) belonged to clade 5a.2a.1. Within clade 5a.2a, 728 (67%) viruses formed a subgroup with T120A and additionally K169Q or V47I (within subclade C.1 [4]). Within clade 5a.2a.1, 710 (97%) viruses formed the C.1.1.1 subclade, defined by T216A and represented by A/Victoria/4897/2022, the virus component for the 2023/24 NH egg-based vaccine (Table 1). In Supplementary Figure S1 we provide the phylogenetic tree of influenza A(H1N1)pdm09 HA genes. A total of 317 (43%) of viruses within clade 5a.2a.1 carried R113K (within C.1.1.1) with or without S85P and 243 (33%) R45K (within C.1.1.1). The different (sub)clades circulated across the reporting weeks and countries.

**Figure 2:**
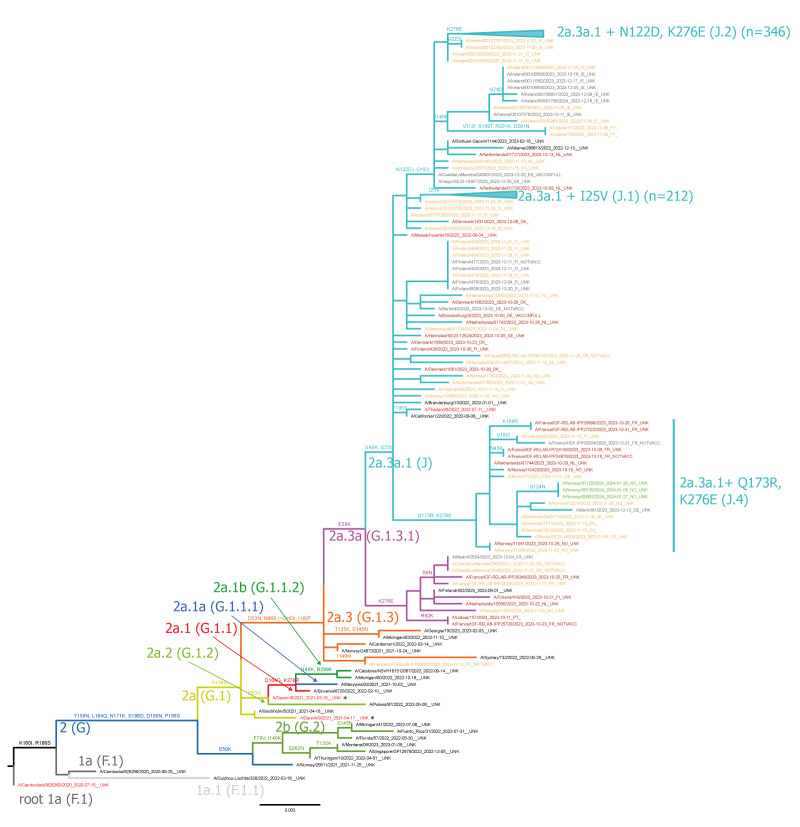
Phylogenetic comparison of influenza A(H3N2) haemagglutinin genes, EU/EEA, weeks 40/2023–9/2024 (n = 639)

EEA: European Economic Area; EU: European Union.

All 639 influenza A(H3N2) viruses with available sequences belonged to clade 2a.3, a subclade of 2a represented by A/Darwin/9/2021, the recommended vaccine strain for egg-based vaccines for 2023/24 NH influenza season (Figure 2). Within clade 2a.3, 98% of isolates (n = 628) were clade 2a.3a.1, represented by A/Thailand/8/2022 which has been recommended in the southern hemisphere (SH) 2024 [10] and the NH 2024/25 [1] vaccine. Most (n = 346; 55%) influenza A(H3N2) isolates in 2a.3a.1 belonged to the J.2 subclade defined by the amino acid substitutions N122D (potential loss of glycosylation site, antigenic site A) and K276E (in antigenic site C). Within 2a.3a.1, a smaller subclade J.1 with I25V (n = 212; 34%) was present (Table 1, Figure 2). The different (sub)clades circulated across the reporting weeks and countries.

The vaccine strains are bright red, reference strains black and sequences reported to TESSy coloured according to the virus collection date by month (2023: October brownish red, November yellow, December grey; 2024: January green, February, turquoise). Clades (subclades) are indicated on the branches or pointed by arrows.

All 90 influenza B/Victoria viruses with available sequences belonged to clade V1A.3a.2 subclade C.5, represented by B/Austria/1359417/2021, the recommended vaccine virus strain for the 2023/24 NH influenza season (Table 1). However, C.5 viruses further diversified, with 39% (n = 35) subclade C.5.1, 32% (n = 29) subclade C.5.7 and 16% (n = 14) subclade C.5.6. A phylogenetic tree of B/Victoria viruses is appended in Supplementary Figure S2. Few viruses represented subclades C.2 (n = 2) and C.3 (n = 1). The different (sub)clades circulated across the reporting weeks and countries.

### Antigenic characterisation

Antigenic characterisation data based on haemagglutination inhibition (HI) from eight countries were available for 675 viruses (Table 1). Germany contributed 77% (n = 523) of the antigenic data, followed by France with 12% (n = 79) and the other countries reporting between <1% and 5%.

Of the 512 characterised influenza A(H1)pdm09 viruses, more than half (n = 262; 51%) were A/Sydney/5/2021-like, 248 (48%) were similar to the vaccine virus A/Victoria/4897/2022-like virus, and two were reported as A/Wisconsin/67/2022-like viruses. The majority of the 103 antigenically characterised A(H3) viruses (n = 76; 74%) were reported as A/Darwin/9/2021-like, 24 (23%) as A/Thailand/8/2022-like, and three were not attributed to any of the reporting categories.

Among 60 antigenically characterised influenza B/Victoria viruses, the majority (n = 58) were similar to the recommended vaccine virus for the 2023/24 NH influenza season (B/Austria/1359417/2021). Two B/Victoria viruses were not attributed to any of the reporting categories (Table 1).

### Antiviral susceptibility

From the beginning of the season until week 9/2024, 2,003 viruses were assessed for antiviral susceptibility to oseltamivir and zanamivir (87% by genomic analysis and 13% by phenotypic analysis) and 1,553 viruses for susceptibility to baloxavir marboxil (all by genomic analyses) in 14 EU/EEA countries (Table 2). In total, five viruses had reduced or highly reduced inhibition or susceptibility based on genetic analyses: three influenza A(H1)pdm09 viruses carried genetic markers associated with either reduced (NA:I223T) or highly reduced inhibition (NA:H275Y) by oseltamivir (Table 2). In addition, 16 influenza A(H1N1)pdm09 viruses carrying amino acid substitutions I223V and S247N in NA were reported by four countries (France, the Netherlands, Norway and Spain). Although no phenotypic data have been reported for these viruses in TESSy, the combination of these two substitutions has been recently shown to be associated with phenotypic reduction in susceptibility [11].

**Table 2:** Influenza subtypes and lineages with and without reduced inhibition following antiviral susceptibility testing to oseltamivir reported to TESSy, EU/EEA, weeks 40/2023 through 9/2024 (n = 2,176)

	Level of susceptibility								
	n	%	n	%	n	%	n	%	
Oseltamivir susceptibility	NI		AANI		AAHRI		AARI		Total
**Influenza A**									
A(H1)pdm09	185	13.0	1,239	86.8	2	0.1^a^	1^b^	0.1	1,427
A(H3)	54	10.5	458	89.5	0		0		512
**Influenza B**									
B/Victoria lineage	21	32.8	43	67.2	0		0		64
**Total**	**260**	**13.0**	**1,740**	**86.9**	**2**	**0.1**	**1**	**0**	**2,003**
Zanamivir susceptibility	NI		AANI		AAHRI		AARI		Total
**Influenza A**									
A(H1)pdm09	185	13.0	1,242	87.0	0		0		1,427
A(H3)	54	10.5	458	89.5	0		0		512
**Influenza B**									
B/Victoria lineage	21	32.8	43	67.2	0		0		64
**Total**	**260**	**13.0**	**1,743**	**87.0**	**0**		**0**		**2,003**
Baloxavir marboxil susceptibility					AANS		AARS		Total
**Influenza A**									
A(H1)pdm09					1,113	100.0	0		1,113
A(H3)					400	99.5	2	0.5^c^	402
**Influenza B**									
B/Victoria lineage					38	100.0	0		38
**Total**					**1,551**	**100.0**	**2**	**0.1**	**1,553**

AA: amino acid (refers to genotypic testing result); EEA: European Economic Area; EU: European Union; NI: normal inhibition; NS: normal susceptibility; HRI: highly reduced inhibition; RI: reduced inhibition; RS: reduced susceptibility; TESSy: The European Surveillance System.

^a^ These viruses carried the mutation NA:H275Y.

^b^ This virus carried the mutation NA:I223T.

^c^ These viruses carried the mutation PA:L28P.

Two influenza A(H3) viruses carried amino acid substitutions associated with reduced susceptibility to baloxavir marboxil (PA:L28P) (Table 2).

All 768 influenza A viruses assessed by genomic analyses for M2 blocker susceptibility showed highly reduced inhibition to adamantanes (influenza B viruses are not susceptible to adamantanes) caused by an S31N amino acid substitution in M2.

## Discussion

Based on our dataset, the 2023/24 influenza season was characterised by co-circulation of influenza A(H1N1)pdm09, A(H3N2) subtypes and B/Victoria-lineage viruses, with A(H1N1)pdm09 being the predominant virus overall in the EU/EEA. This was in contrast to the previous season, when influenza A(H3) viruses predominated [12], even though variation between countries occurred.

Regarding the antigenic similarity to the 2023/24 NH vaccine component (A/Victoria/4897/2022-like clade 5a.2a.1 virus (egg-based)), the circulating influenza A(H1N1)pdm09 viruses appeared to be overall antigenically similar. Interim European vaccine effectiveness data, September 2023 to January 2024, showed 53% (95% confidence interval (CI): 41–63) protection against influenza A(H1N1)pdm09 in all ages in primary care and 44% (95% CI: 30–55) for hospitalised patients [13]. Some genetic diversification was observed in the 5a.2a viruses, with branches having defined amino acid substitutions with a significant number of viruses such as T120A with K169Q or V47I in 5a.2a and R113K ± S85Pand R45K in 5a.2a.1. Despite the genetic heterogeneity of recently circulating influenza A(H1)pdm09 viruses, the WHO recommended to retain the 2023/24 A(H1)pdm09 component for the 2024/25 influenza season, based on human serology study results confirming that the NH 2023/24 vaccine post-vaccination serum titres were not substantially reduced for most circulating viruses [1]. When comparing the circulation patterns of influenza A(H1N1)pdm09 viruses in Europe with the ones circulating globally through Nextstrain real-time tracking of influenza virus evolution for the same reporting period as our analysis [6], we noticed similar patterns of subclades circulating in North and South America, Australia and Asia. Lower vaccine effectiveness (VE) against clade 5a.2a.1 viruses (39%; 95%the CI: −44 to 74) compared with clade 5a.2a viruses (52%; 95% CI: −7 to 78) was, however, observed in the European interim VE study [13]; this is also supported by the mid-season Canadian Sentinel Practitioner Surveillance Network VE studies (56% (95% CI: 33–71) for 5a.2a.1 vs 67% (95% CI: 48–80) for clade 5a.2a) [14]. Reduced inhibition by oseltamivir was detected in only three A(H1)pdm09 viruses, with the large majority of tested viruses remaining susceptible.

For influenza A(H3N2), almost all circulating viruses fell genetically in clade 2a.3a.1, represented by influenza A/Thailand/8/2022, which has recently been recommended as the vaccine component for the NH 2024/25 influenza season [1]. In serology studies using post-vaccination human sera, reduced HI reactivity was seen against some recent viruses expressing HA genes from subclade 2a.3a.1 [1]. Notably, in the EU/EEA, more than half (n = 346; 54%) of influenza A(H3N2) viruses belonged genetically to this divergent clade 2a.3a.1 with additional amino acid substitutions in the antigenic sites N122D and K276E (J.2), and another subgroup with I25V (J.1). When comparing the circulation patterns of influenza A(H3N2) viruses in Europe to the ones circulating globally [6], we noticed similar patterns of subclades circulating globally, with J.2 viruses dominating and co-circulation of J.1 and other J clade viruses. Interim European VE results in primary care for all ages indeed showed a reduced protection for currently circulating influenza A(H3N2) viruses of 30% (95% CI: −3 to 54) and, in hospital studies, 14% (95% CI: −32 to 43). This indicated that many of the currently circulating 2a.3a.1 subclade strains in the EU/EEA had diversified antigenically from the NH 2023/24 vaccine virus A/Darwin/9/2021 [1]. In our EU/EEA data, only 23% of antigenically characterised viruses were A/Thailand/8/2022-like which would indicate that they were less well recognised by the vaccine virus A/Darwin/9/2021 antisera. However, it needs to be noted that antigenic characterisations were not performed for all circulating 2a.3a.1 viruses, that not all laboratories would have had potentially both A/Darwin/9/2021 and A/Thailand/8/2022 antisera and that antigenic characterisation data do not necessarily reflect the proportion of different (sub)clades among circulating viruses. Furthermore, differences in the antigenic and/or human serology data in comparison with VE data could possibly arise from the fact that in the EU/EEA, as elsewhere, most available vaccines are produced in eggs rather than in cell lines [15-18]. Reduced susceptibility to baloxavir marboxil was reported in only two influenza A(H3) viruses from two different countries, while the large majority of tested viruses remained susceptible.

For the B/Victoria lineage, all antigenically characterised viruses were V1A.3a.2 B/Austria/1359417/2021-like, which is the current vaccine component in tri- and quadrivalent vaccines in the NH 2023/24 season. Even if genetic diversification continues within this lineage, the currently circulating viruses in the EU/EEA have still been well covered by the vaccine virus antigenically, and the WHO did not propose an update to the vaccine component [1]. When comparing the circulation patterns of B/Victoria viruses in Europe to the ones circulating globally [6], we noticed similar patterns of subclades circulating in Asia, Australia and in South Africa, with C.5.7 viruses dominating. However, C.5.1 viruses dominated in North America and C.5.4 viruses in South America.

There are some additional limitations to these data. The specimen sources (sentinel general practitioners, hospitals, intensive care units, outbreak investigations) and selection processes for the viruses that undergo characterisation vary from country to country and over time. Only a small percentage (0.4% antigenically and 2% genetically; 3% and 6% of the sentinel source viruses, respectively) of detected viruses from a limited number of countries were characterised overall, and the contributions of countries to different datasets varied. The ECDC and WHO Regional Office for Europe have previously recommended sequencing all influenza viruses detected from sentinel sources, and we are still far from this target [3]. This is partly due to the preselection of specimens based on the PCR quantification cycle value to ensure that selected specimens have a sufficient viral load for sequencing. Furthermore, the panel of antisera used by laboratories for antigenic characterisation does deliberately not include all circulating clades as some clades are antigenically similar (see Methods).

## Conclusion

Despite the challenges in collecting influenza surveillance data, detection and characterisation of influenza viruses within the EU/EEA play a vital role in identifying which viruses should be sent to a WHO Collaborating Centre for in-depth analysis. These analyses are essential for guiding the decision-making process during the semiannual WHO influenza vaccine composition meetings.

## Collaborators’ affiliations

Belgium: Sarah Denayer, National Influenza Center in Belgium, Sciensano, Brussels; François E. Dufrasne, National Influenza Center in Belgium, Sciensano, Brussels.

Bulgaria: Neli Korsun and Ivelina Trifonova, National Centre of Infectious and Parasitic Diseases, Sofia.

Croatia: Goranka Petrović and Irena Tabain, Croatian Institute of Public Health, Zagreb.

Czechia: Helena Jiřincová and Timotej Šúri, National Institute of Public Health, Prague.

Denmark: Amanda Bolt Botnen and Ramona Trebbien, Statens Serum Institut, Copenhagen.

Estonia: Johanna Kristina Tamm and Regina Russanova, Communicable Diseases Laboratory, Health Board, Tallinn.

Finland: Niina Ikonen and Erika Lindh, Finnish Institute for Health and Welfare (THL), Helsinki.

France: Vincent Enouf, National Reference Center of Respiratory Viruses - Institut Pasteur, Paris; and Laurence Josset, National Reference Center of Respiratory Viruses - Hospices Civils de Lyon, Lyon.

Germany: Marianne Wedde and Ralf Dürrwald, Robert Koch Institute, Berlin.

Greece: Georgia Gioula, National influenza Centre for Northern Greece, Thessaloniki and Emmanouil Mary, National Reference Laboratory for Southern Greece, Hellenic Pasteur Institute, Athens.

Iceland: Brynja Ármannsdóttir, Landspitali and Guðrún Erna Baldvinsdóttir, Landspitali – The University Hospital of Iceland, Reykjavík.

Italy: Simona Puzelli and Marzia Facchini, Institute of Health (Istituto Superiore di Sanità), Rome.

Lithuania: Svajune Muralyte and Monika Maconkaite-Tekoriene, National Public Health Surveillance Laboratory, Vilnius.

Luxembourg: Anke Wienecke-Baldacchino and Trung Nguyen, Laboratoire national de santé, Diddeléng.

Malta: Dr Graziella Zahra and Dr Jackie Melillo, Health Promotion and Disease Prevention, Department for Health Regulation, Ministry for Health and Active ageing, Pieta.

The Netherlands: Adam Meijer, Dutch National Institute for Public Health and the Environment (RIVM), Bilthoven and Ron Fouchier, Erasmus University Medical Center, Rotterdam.

Norway: Andreas Rohringer and Karoline Bragstad, Norwegian Institute of Public Health, Oslo.

Poland: Lidia B. Brydak, Ewelina Hallmann, Laboratory of Influenza Viruses and Respiratory Viruses, Department of Virology, National Institute of Public Health NIH – National Research Institute, Warsaw.

Portugal: Raquel Guiomar, National Reference Laboratory for Influenza and Other Respiratory Viruses, Infectious Diseases Department, and Ana Paula Rodrigues, Department of Epidemiology, National Institute of Health Doctor Ricardo Jorge, Lisbon.

Romania: Mihaela Lazar, National Influenza Centre, “Cantacuzino” National Military-Medical Institute for Research and Development, Rodica Popescu, National Institute of Public Health Romania, Bucharest.

Slovakia: Edita Staroňová, National Influenza Center, Public Health Authority of the Slovak republic, Bratislava and Elena Tichá, National Influenza Center, Public Health Authority of the Slovak republic, Bratislava.

Slovenia: Vesna Šubelj and Katarina Prosenc, National Laboratory for Health, Environment and Food, Ljubljana.

Spain: Francisco Pozo and Inmaculada Casas, National Centre for Microbiology, Institute of Health Carlos III, Madrid and Consortium for Biomedical Research in Epidemiology and Public Health (CIBERESP).

Sweden: Tove Samuelsson Hagey and Neus Latorre-Margalef, Public Health Agency of Sweden, Stockholm.

## Supplementary Data

1221000 Supplement
