# Supplementary material for: Antigenic changes in influenza A(H3N2) driven by genetic evolution: Insights from virological surveillance, EU/EEA, week 40/2023 to week 9/2024
Source: Euro Surveill. 2024 Dec 12;29(50):2400395. doi: 10.2807/1560-7917.ES.2024.29.50.2400395 (PMC11650508; doi:10.2807/1560-7917.ES.2024.29.50.2400395)

This supplementary material is hosted by Eurosurveillance as supporting information alongside the article Broberg et al. Antigenic changes in influenza A(H3N2) driven by genetic evolution: Insights from virological surveillance, EU/EEA, week 40/2023 to week 9/2024, on behalf of the authors, who remain responsible for the accuracy and appropriateness of the content. The same standards for ethics, copyright, attributions and permissions as for the article apply. Supplements are not edited by Eurosurveillance and the journal is not responsible for the maintenance of any links or email addresses provided therein.

Supplemental materials

Supplemental Figure 1 S1. Phylogenetic comparison of influenza A(H1N1)pdm09 haemagglutinin genes. The vaccine strains are red, reference strains black and sequences reported to TESSy coloured according to the virus collection date by month (2023: October red, November yellow, December grey; 2024: January green, February, turquoise).

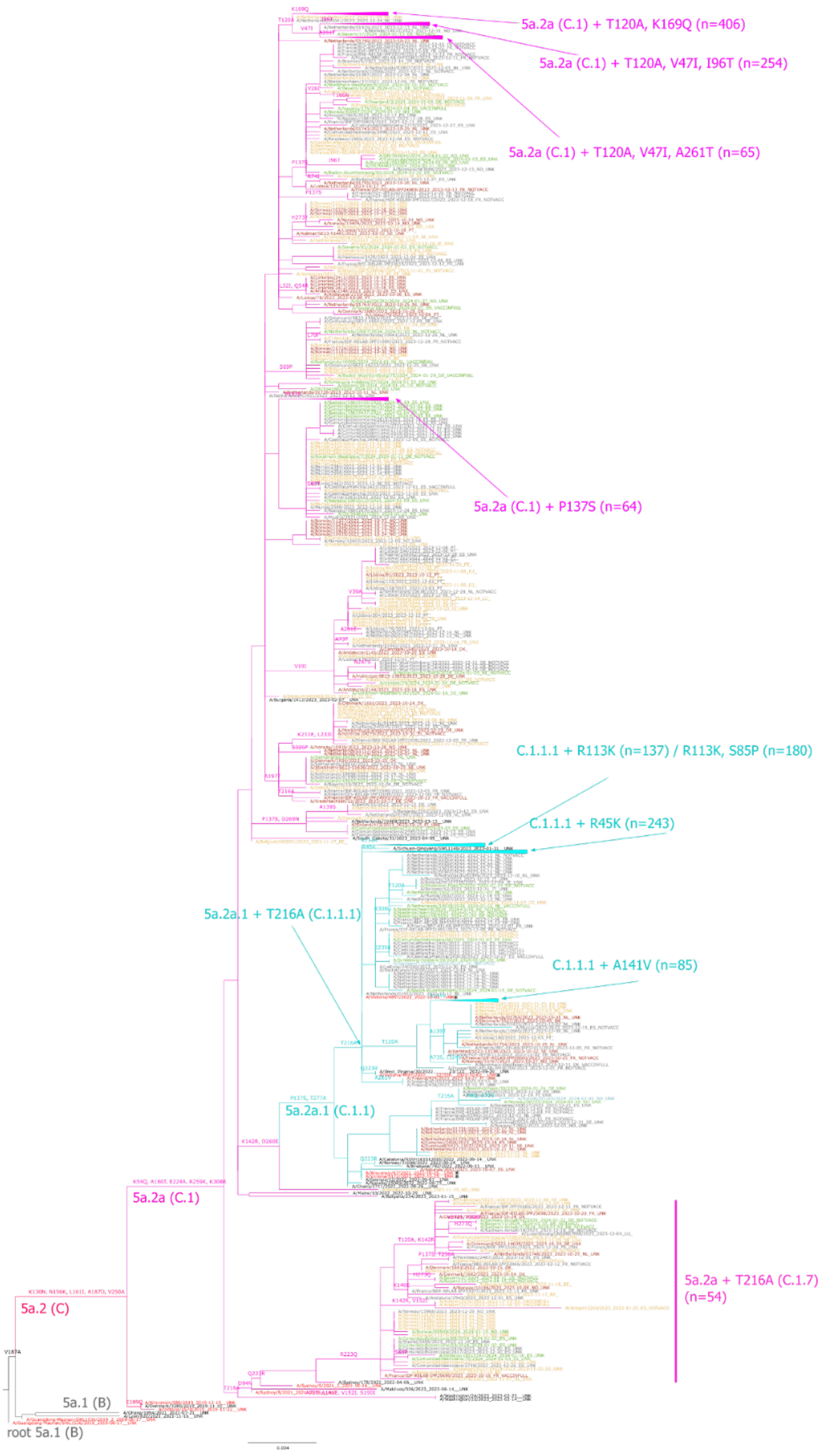

Supplemental Figure 2 S2. Phylogenetic comparison of influenza B/Victoria-lineage haemagglutinin genes. The vaccine strains are red, reference strains black and sequences reported to TESSy coloured according to the virus collection date by month (2023: October red, November yellow, December grey; 2024: January green, February, turquoise).

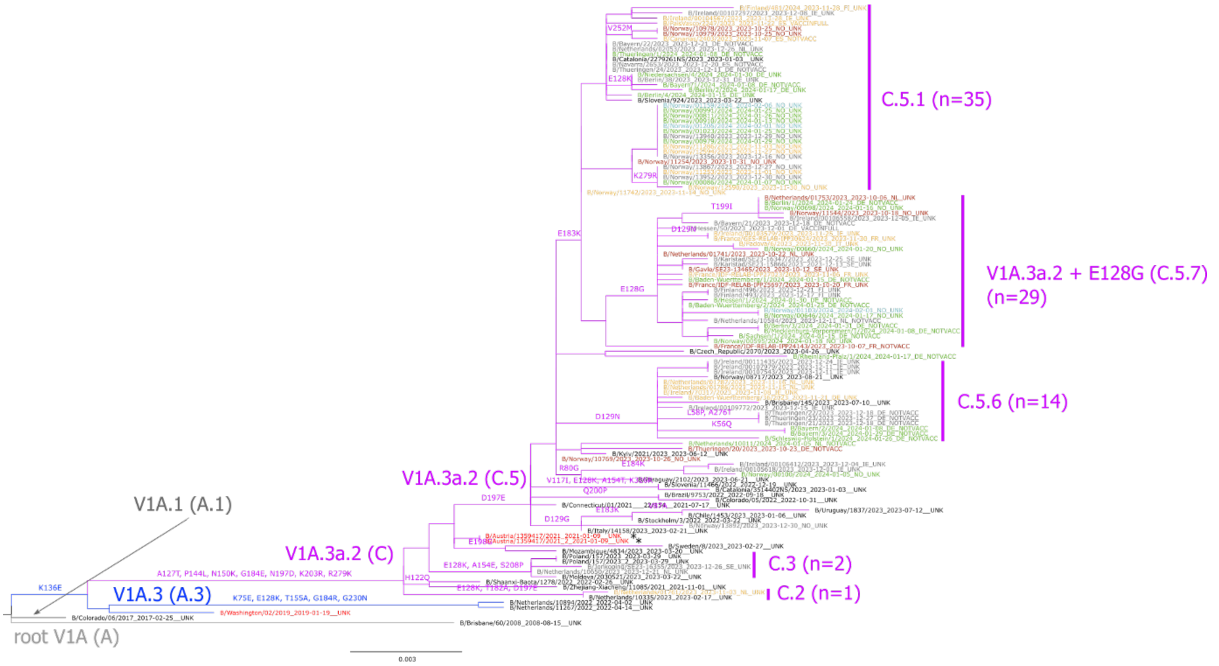

Supplement: Supplement [file 24-00395_BROBERG_Supplement.pdf]
